# Study on βTCP/P(3HB) Scaffolds—Physicochemical Properties and Biological Performance in Low Oxygen Concentration

**DOI:** 10.3390/ijms231911587

**Published:** 2022-09-30

**Authors:** Szymon Skibiński, Joanna P. Czechowska, Ewelina Cichoń, Martyna Seta, Agata Gondek, Agnieszka Cudnoch-Jędrzejewska, Anna Ślósarczyk, Maciej Guzik, Aneta Zima

**Affiliations:** 1 Faculty of Materials Science and Ceramics, AGH University of Science and Technology, Mickiewicza 30, 30-059 Krakow, Poland; 2 Chair and Department of Experimental and Clinical Physiology, Medical University of Warsaw, Banacha 1b, 02-091 Warsaw, Poland; 3 Department of Methodology, Medical University of Warsaw, Banacha 1b, 02-091 Warsaw, Poland; 4 Jerzy Haber Institute of Catalysis and Surface Chemistry Polish Academy of Sciences, Niezapominajek 8, 30-239 Krakow, Poland

**Keywords:** biomaterials, β tricalcium phosphate, poly(3-hydroxybutyrate), polyhydroxyalkanoates, scaffolds, bone tissue engineering, in vitro studies, mesenchymal stem cells

## Abstract

The search for new materials for bone regenerative purposes is still ongoing. Therefore, we present a series of newly constructed composites based on β tricalcium phosphate (βTCP) and poly(3-hydroxybutyrate) bacteria-derived biopolymer (P(3HB)) in the form of 3D scaffolds with different pore sizes. To improve the polymer attachment to the βTCP surface, the etching of ceramic sinters, using citric acid, was applied. As expected, pre-treatment led to the increase in surface roughness and the creation of micropores facilitating polymer adhesion. In this way, the durability and compressive strength of the ceramic–polymer scaffolds were enhanced. It was confirmed that P(3HB) degrades to 3-hydroxybutyric acid, which broadens applications of developed materials in bone tissue engineering as this compound can potentially nourish surrounding tissues and reduce osteoporosis. Moreover, to the best of our knowledge, it is one of the first studies where the impact of βTCP/P(3HB) scaffolds on mesenchymal stem cells (MSCs), cultured in lowered (5%) oxygen concentration, was assessed. It was decided to use a 5% oxygen concentration in the culture to mimic the conditions that would be found in damaged bone in a living organism during regeneration. Scaffolds enabled cell migration and sufficient flow of the culture medium, ensuring high cell viability. Furthermore, in composites with etched βTCP, the MSCs adhesion was facilitated by hydrophilic ceramic protrusions which reduced hydrophobicity. The developed materials are potential candidates for bone tissue regeneration. Nevertheless, to confirm this hypothesis, in vivo studies should be performed.

## 1. Introduction

Despite remarkable progress in the field of regenerative medicine, there are still many clinical challenges in the effective treatment of bone defects. Although autografts are still considered the “gold standard” in bone transplantation, both the limited amount of harvested tissue material and the risk of postoperative complications are barriers [1]. Therefore, a dynamically growing area of research is tissue engineering, where one of the challenges is to develop artificial scaffolds supporting the regeneration of damaged tissues or even whole organs.

Calcium phosphates (CaPs), due to their similarity in terms of chemical composition to the main component of the inorganic part of bone, exquisitely support bone tissue regeneration by releasing calcium and phosphate ions required for its mineralisation [2]. The Ca/P ratio and crystallinity of CaP influence the performance of this process. However, it should be noted that CaP scaffolds are meant to serve as substrates that will resorb and be subsequently replaced by growing bone tissue over time. Hydroxyapatite materials resorb too slowly and α-tricalcium phosphate-based materials resorb too quickly concerning the rate of bone reconstruction [3]. Therefore, in this work, we decided to use β tricalcium phosphate (βTCP), which exhibits a suitable resorption rate for tissue engineering applications [4].

Nevertheless, bioceramic scaffolds exhibit low compressive strength and high brittleness. The fabrication of ceramic–polymer composites using degradable polymers enables not only the improvement of mechanical properties [5] but also their potential use as carriers of biologically active substances supporting tissue regeneration [6,7]. Several studies have focused on the use of biopolymers in the preparation of biomaterials. For example, chitosan, gelatine, zein, or polycaprolactone were exploited due to their ability to further enhance the biomaterial performance and also adequately modulate desired properties for tailor-made applications [8,9]. Similarly, bacteria-derived biopolymers were reported to be suitable for the creation of biomedical materials. One example of such microbial polyesters is polyhydroxyalkanoates (PHAs) [10]. PHAs are of special interest as they are biocompatible and biodegradable materials, and their degradation products ((*R*)-3-hydroxy acids) can potentially nourish surrounding tissues. To date, however, most research has focused on the potential use of PHAs in soft tissue regeneration [11,12]. Poly(3-hydroxybutyrate) (P(3HB)) belongs to short-chain length PHA and it is considered a promising biopolymer with biomedical applications like the development of stents, orthopaedic pins, sutures, or cardiovascular patches [13]. P(3HB) as a coating in inorganic-organic composite was applied in bioglass-TiO_2_ scaffolds, where the polymeric layer was enriched with chitosan, and carbon nanotubes resulting in enhanced bioactivity [14]. It has been reported that the P(3HB) improved the mechanical strength and bioactivity of glass–ceramic scaffolds, however, the polymeric layer was not homogeneous with the presence of uncoated surface regions [15]. To enhance polymer adhesion, many techniques such as mechanical roughening, silane treatment, laser treatment, and flame treatment have been used to modify ceramic surfaces [16]. The promising method is acid etching, which is applied to create roughness and pores in order to improve the polymer attachment to the biomaterial.

The important features of scaffold design for bone tissue engineering are pore sizes and architecture. According to the literature, the average pore size of ~550 μm is an optimal size for bone tissue overgrowth [17]. However, other studies demonstrated faster osteogenesis and angiogenesis in vivo on CaP scaffolds with pore sizes between 750–900 μm [18,19]. In contrast, several studies have demonstrated an important role of micropores (<50 µm) in bone tissue formation [20,21]. Therefore, there is still a necessity in exploring the interactions between the cells and scaffolds with different pore architecture (size, geometry, and interconnectivity of pores).

Knowing that the Mesenchymal Stem Cells (MSCs) have the potential to differentiate into *inter alia*, osteocytes, chondrocytes, and adipocytes, they are the stem cells of choice to check if scaffolds are well designed for bone tissue regeneration purposes. Moreover, MSCs can affect the action of the immune system through interactions with cells of both the innate and adaptive immune systems. The literature data also showed that MSC can reduce the release of pro-inflammatory cytokines at the site of injury or damaged tissue and protect cells in damaged areas from apoptosis. Their important role in tissue regeneration and repair processes, as well as in angiogenesis, was also indicated [22,23,24]. In addition, the fact that MSCs have been studied for more than 30 years and are used in more than 1130 clinical trials, is not to be underestimated [25]. Further, the MSC culture under reduced (physiological) oxygen conditions was used because the literature and our own research show that application in cell culture physiological normoxia results in faster stem cells division, longer maintenance of cellular stemness in culture (maintenance of expression of markers typical of stem cells and mesenchymal stem cells), better cell condition, and slower ageing [25,26,27,28,29]. In addition, these conditions are more similar to those of tissues in the human body (about 2–7%) than the 21% (ambient) oxygen concentration used in standard cell cultures. Moreover, many studies show the effect of lowered oxygen concentration on MSC differentiation. These studies show that a lowered oxygen concentration in culture promotes the differentiation of MSCs into chondrocytes [28,30] and that a 5% oxygen concentration enhanced osteogenic potential [31,32]. Thus, in our study, it was decided to use a 5% oxygen concentration in the culture to better replicate the conditions that would be found in damaged bone in a living organism during regeneration [33].

In this study, porous βTCP and composite βTCP/P(3HB) scaffolds with different pore sizes were developed by combining the polyurethane sponge replica and the polymer infiltration methods. In order to enhance the polymer attachment to the βTCP surface, pre-treatment of the ceramic component was performed. The influence of the different pore sizes of the scaffolds, ceramic sinters pre-treatment, as well as polymeric coating on the physicochemical and biological properties were evaluated. To the best of our knowledge, it is one of the first studies where the impact of βTCP/P(3HB) scaffolds on human bone marrow-derived MSCs, cultured in lowered (5%) oxygen concentration, was assessed.

## 2. Results and Discussion

### 2.1. Phase Composition

**Figure 1:**
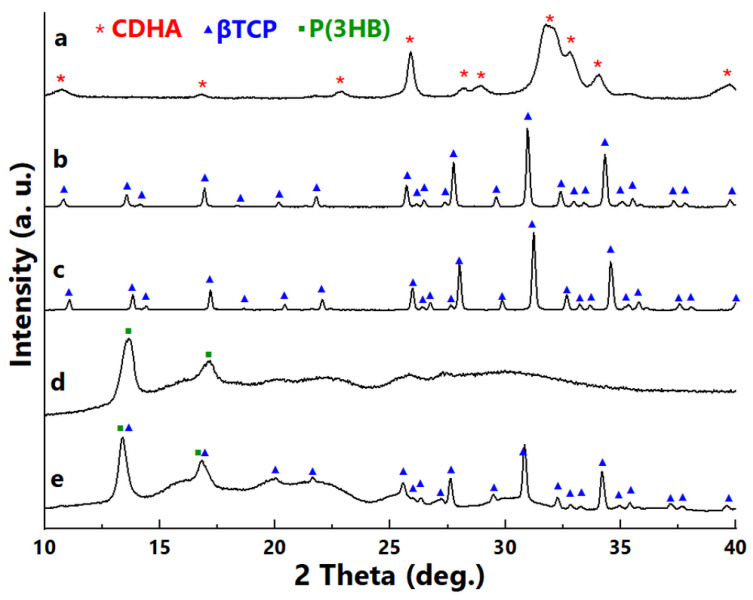
XRD patterns of: (**a**) TCP powder before calcination, (**b**) βTCP powder after calcination, (**c**) TM scaffold, (**d**) P(3HB) polymer, (**e**) composite βTCP/P(3HB) disc.

The XRD analysis revealed that semi-crystalline calcium-deficient hydroxyapatite (CDHA) was present in the TCP powder before calcination (Figure 1a). The pure βTCP (Figure 1b) was obtained by thermal conversion (2 h at a maximum temperature of 900 °C) of CDHA according to the reaction presented in Equation (1).

Ca_9_(HPO_4_)(PO_4_)_5_(OH) → 3Ca_3_(PO_4_)_2_ + H_2_O (1)

These results are compliant with Bakan [34], who demonstrated that the full transformation from CDHA to βTCP for the powders synthesized under a 5.5 pH value is possible at 750 °C. The occurrence of one crystalline phase—βTCP was also identified in the phase composition of the sintered bioceramic scaffolds (TS, TM, TL) at 1150 °C (Figure 1c presenting diffractogram of TM). Diffractogram of P(3HB) (Figure 1d) was typical for semi-crystalline PHA, with clearly visible peaks at 2θ = 13.62° and 17.19° corresponding to orthorhombic crystal planes (020), (110). These peaks were also observed by Anbukarasu et al. [35] at 2θ values of 13.5° and 16.85°. In the case of composite, the presence of the polymer layer was confirmed—the reflexes originating from both βTCP and P(3HB) were noticed (Figure 1e).

### 2.2. Thermal Analysis

**Figure 2:**
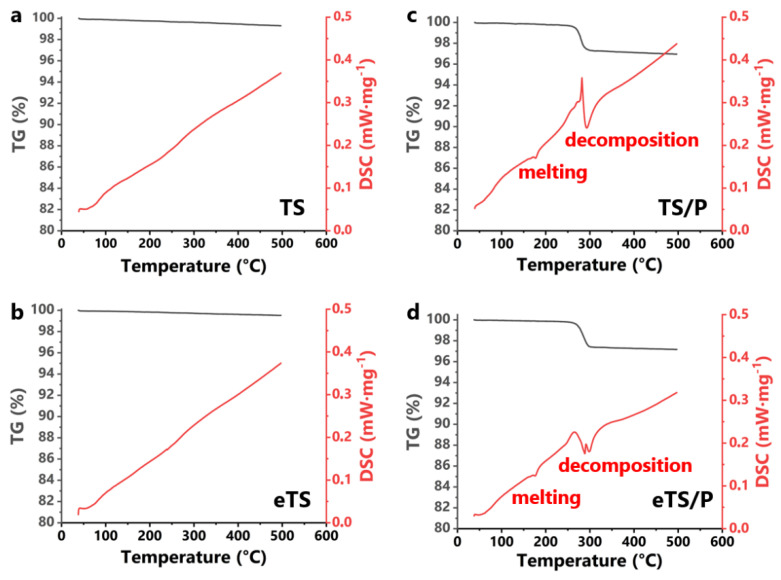
TG-DSC curves of (**a**) TS, (**b**) eTS, (**c**) TS/P, and (**d**) eTS/P scaffolds.

Thermogravimetric (TG) and differential scanning calorimetry (DSC) curves of TS, eTS, TS/P, and eTS/P are presented in Figure 2. The TG curves of TS and eTS show only slight weight loss (0.7 wt% and 0.5 wt%, respectively), which may be attributed to the evaporation of absorbed water. The polymer content in the scaffolds, determined *via* the TG method, was equal to 3.0 wt% and 2.8 wt% for TS/P and eTS/P, respectively. In the case of the bioceramic materials, neither peaks corresponding to endothermic nor exothermic reactions were observed in the endotherms. Contrarily, TS/P and eTS/P composites display a weight loss starting at 268 °C and ending at 287 °C, leading to P(3HB) decomposition. DSC curves of the composites exhibit sharp peaks at 176.6 °C and 287.1 °C for TS/P and at 177.0 °C and 284.3 °C for eTS/P. The observed endothermic reactions correspond to the P(3HB) melting point and its decomposition, respectively [36].

### 2.3. Microstructure and Porosity

**Figure 3:**
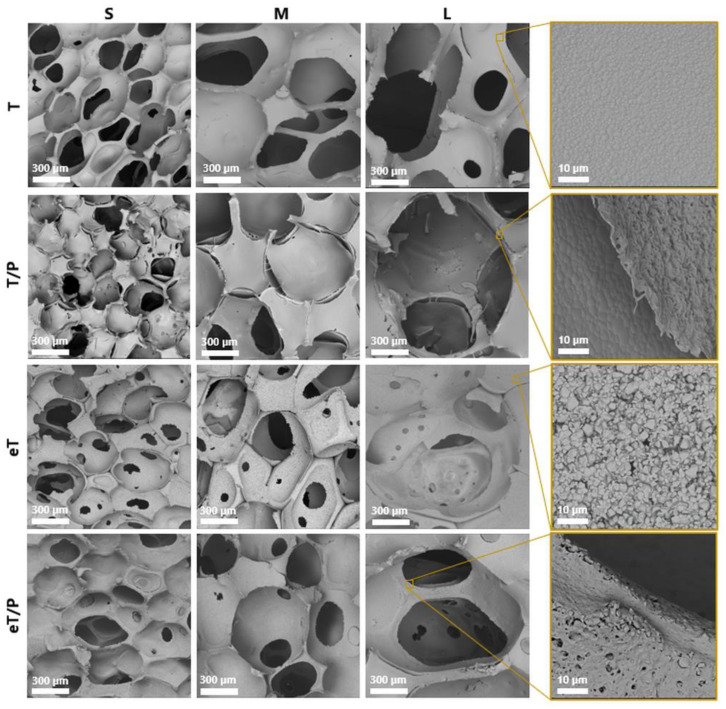
Microstructure of the obtained scaffolds.

The microstructure of the scaffolds is presented in Figure 3. The developed TCP scaffolds exhibited a macroporosity characteristic of the microstructures obtained by the polyurethane sponge replica method [37]. SEM observations showed the non-homogeneous distribution of the polymer layer on the composite scaffolds (Figure 3 T/P). Similar observations were made by Bretcanu et al. [15], who demonstrated that the P(3HB) coating on the glass-ceramic materials was not homogeneous and revealed the presence of uncoated surface regions. In addition, Li et al. [38] noticed that the struts of the 45S5 Bioglass^®^ scaffolds were only partly covered by another short chain length PHA—poly(3-hydroxybutyrate-co-3-hydroxyvalerate) (PHBV).

**Figure 4:**
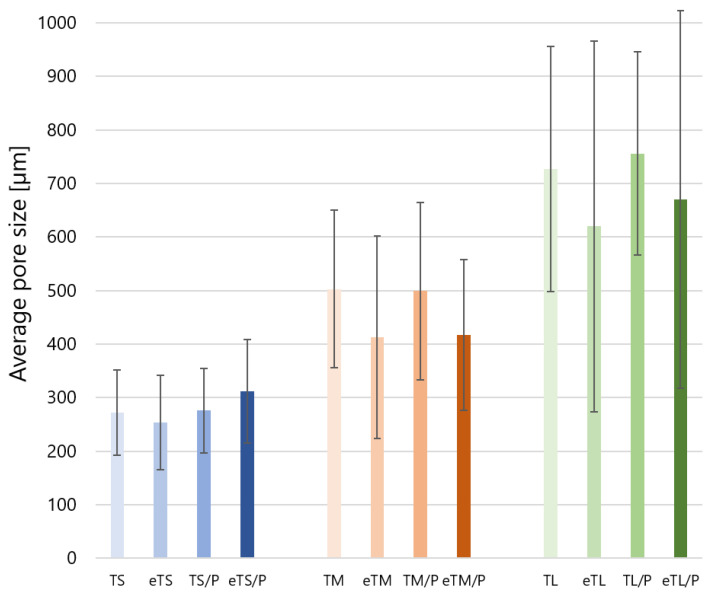
The average pore size of the scaffolds.

Previous studies by Cichoń et al. [5] on mcl-PHA polymer, poly(3-hydroxyoctanoate) P(3HO) also showed the detachment of the polymer from the ceramic surface. However, in the case of P(3HO), the detachment was less visible than on the materials coated with P(3HB). This behaviour is probably due to the physicochemical properties of different PHA polymers: brittle P(3HB) vs. elastomeric P(3HO), which enables better alignment to the ceramic scaffold. In this study, to improve the attachment of the polymer to the βTCP surface, a simple and effective method of etching the ceramic scaffolds with citric acid (CA) was performed. The idea was derived from dental practice where an adequate adhesion between the restoration and restorative material is achieved by the surface treatment with, i.e., hydrofluoric acid [39]. As evidenced by SEM observations (Figure 3 eT), the etching with CA affected the microstructure of the ceramic scaffolds, making their surface rougher. Furthermore, the 3 min exposure to CA resulted in the appearance of micropores (in the range from about 15 to 100 µm), called ‘windows’. The average pore size of the ceramic scaffolds (Figure 4) varied depending on the polyurethane matrix used and was equal to 272 ± 79 µm, 503 ± 147 µm and 727 ± 229 µm for TS, TM and TL, respectively. A slight decrease in the average pore size for etched materials, i.e., eTS (254 ± 88 μm), eTM (413 ± 189 μm), and eTL (620 ± 346 µm), was connected with the presence of ‘windows’. In the case of T/P composites, the polymer layer attached to the ceramic surface did not significantly affect the average pore size of the scaffolds.

**Figure 5:**
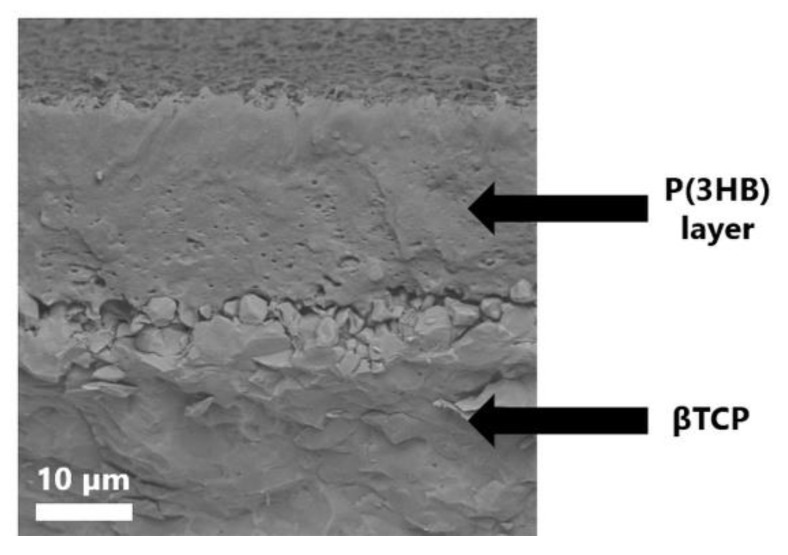
Cross-section of the composite etched βTCP disc covered with P(3HB).

When the etched bioceramic materials were coated with P(3HB), a better attachment at the ceramic/polymer interface was observed than for non-etched scaffolds—the polymer was able to penetrate within the etched roughness of the ceramic surface (Figure 5), thus creating a stronger physical bond between composite compounds.

**Figure 6:**
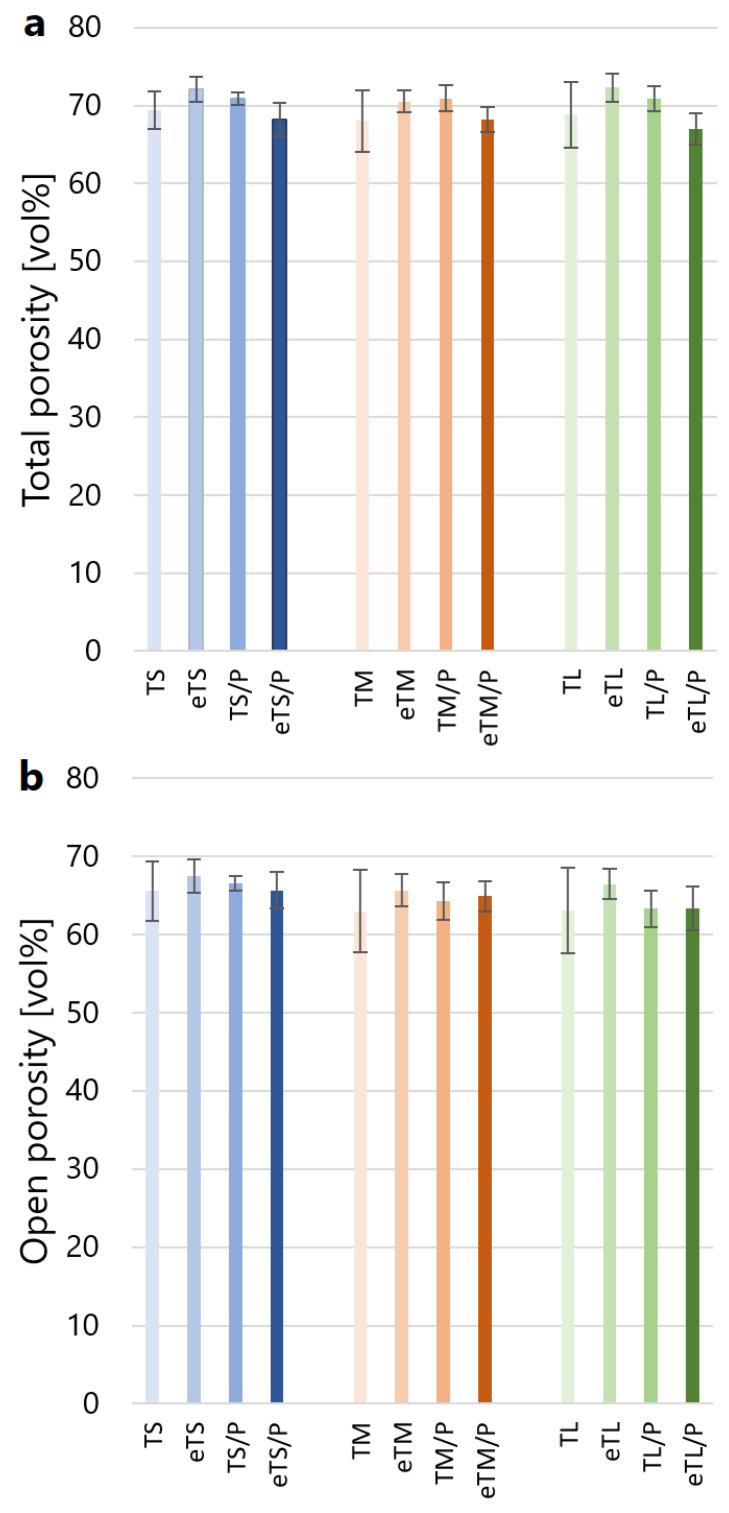
Total (**a**) and open (**b**) porosity of the obtained materials.

This resulted in the improved adhesion of P(3HB) to the scaffolds’ walls and no polymer detachment was observed (Figure 3 eT/P). Polymer coating did not influence the average pore size and was equal to 312 ± 97 µm, 417 ± 141 µm, and 670 ± 352 µm for eTS/P, eTM/P, and eTL/P, respectively. All scaffolds are characterised by the pore sizes favouring osteoinductivity and osteogenesis [17]. The consecutive parameter taken under consideration was the porosity of synthesised materials, which is also an important factor that influences the biological behaviour of scaffolds for bone tissue regeneration. The open porosity (from 63.0 ± 5.3 to 67.5 ± 2.1 vol%) of the obtained bioceramic materials was close to their total porosity (from 68.0 ± 4.0 to 72.3 ± 1.8 vol%), which indicates that the amount of closed pores is negligible (Figure 6). In the case of the composites, the P(3HB) layer did not influence the open (from 63.3 ± 2.4 to 66.6 ± 0.9 vol%) and total (67.0 ± 2.0 to 70.9 ± 1.7 vol%) porosity when compared to the bare βTCP scaffolds. Those values are consistent with the porosity of cancellous bone (50–90 vol%) [40].

### 2.4. Wettability and Surface Free Energy Measurements

**Figure 7:**
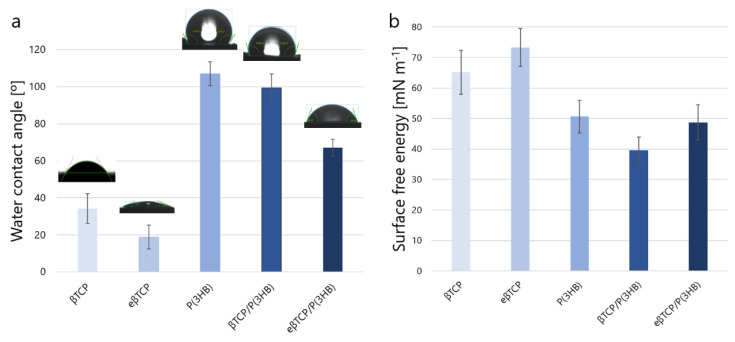
Water contact angle (**a**) and surface free energy (**b**) of the obtained materials.

Wettability and surface free energy are believed to be important parameters that influence biological response to biomaterials. The wettability studies revealed that the coating of βTCP materials significantly changed their character from hydrophilic to hydrophobic, i.e., from 34° ± 8° for βTCP to 99° ± 8° for βTCP/P(3HB). It also decreases the surface free energy of the prepared materials, i.e., from 65 ± 7 mN m^−^^1^ for βTCP to 40 ± 4 mN m^−^^1^ for βTCP/P(3HB). These observations are in line with our previous studies with other polyhydroxyalkanoates, i.e., P(3HO) [5]. Interestingly, in the case of pre-etched polymer-coated ceramic scaffolds, the decrease in water contact angle from 99° ± 8° (βTCP/P(3HB) to 67° ± 5° (eβTCP/P(3HB), Figure 7) was observed. It is due to the increase in the surface roughness that some of the hydrophilic βTCP grains protruded on the eT/P surface, as evidenced by SEM observations (Figure 3). A similar trend was observed for apatite minerals, where authors reported that the increase in surface roughness has a significant impact on increasing wettability [41]. Such modifications may have a positive effect on the biological performance of developed materials. The initial surface roughness and wettability of biomaterials could influence macromolecular biological responses such as the adsorption of plasma proteins [42].

### 2.5. Compressive Strength

**Figure 8:**
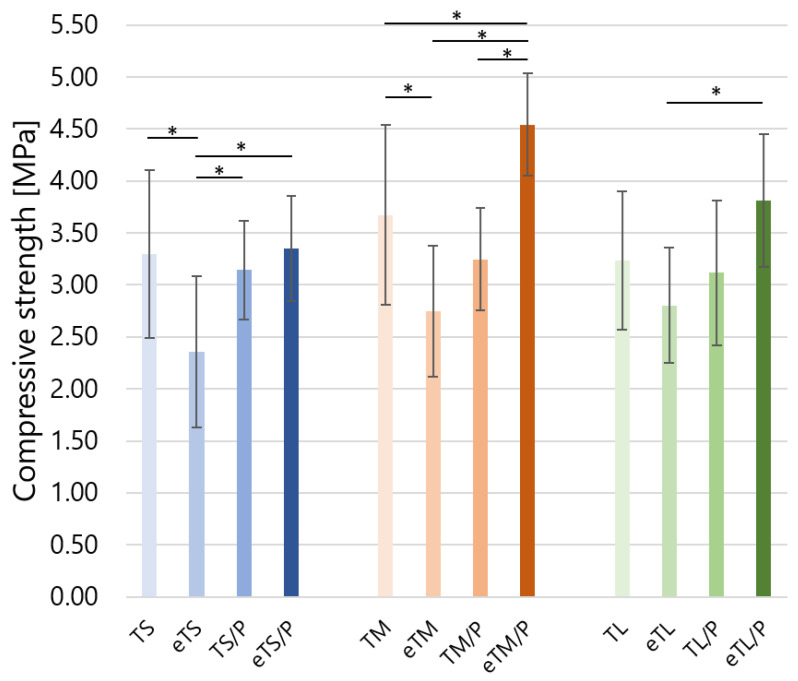
Compressive strength of the scaffolds. Statistically significant differences were indicated by * *p* ≤ 0.01.

The obtained scaffolds were subjected to compressive strength tests (Figure 8). The presence of the polymer and its improved adhesion had a noticeable effect on the mechanical properties and durability of the ceramic–polymer scaffolds. This parameter for the composites with etched βTCP was 3.3 ± 0.5, 4.5 ± 0.5, and 3.8 ± 0.6 MPa (for eTS/P, eTM/P, and eTL/P, respectively) and in the case of eTM/P was higher if compared to their non-etched analogues. The obtained composites meet the mechanical requirements for non-load bearing bone substitutes as the compressive strength of spongy bone varies within 1.5–12.0 MPa [43]. There were no statistically significant differences between the mechanical strength of non-covered (TS, TM, TL) and polymer-covered untreated scaffolds (TS/P, TM/P, TL/P). This was probably due to the weak adhesion between βTCP and P(3HB) and the polymer layer discontinuity. The improvement was noticed in the case of pre-etched composite scaffolds where statistically significant differences were observed. The mechanism increasing the fracture toughness of brittle materials covered by polymers is crack bridging [44]. This phenomenon is responsible for the increase in the compressive strength of pre-etched composites.

**Figure 9:**
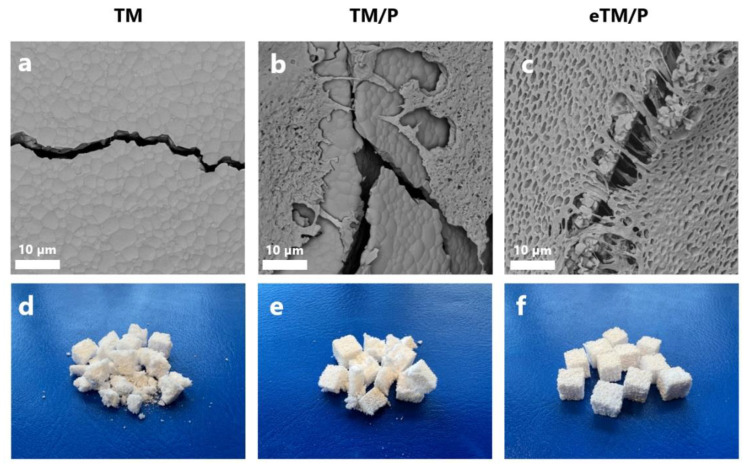
SEM micrographs of the (**a**) TM, (**b**) TM/P, (**c**) eTM/P scaffolds after compression test and macroscopic view of the compressed (**d**) TM, (**e**) TM/P, (**f**) eTM/P specimens.

SEM images of the compressed materials confirmed that the polymer fibers bridge cracks, reinforcing the infiltrated with P(3HB) ceramic scaffolds (Figure 9a–c). The most profound effect of this phenomenon was observed in the etched scaffolds covered with the polymer, which also resulted in the macroscopic durability of the composites (Figure 9e–g). Durability is a desirable feature for implantable materials that will not disintegrate easily.

### 2.6. Chemical Stability and Degradation Studies In Vitro

**Figure 10:**
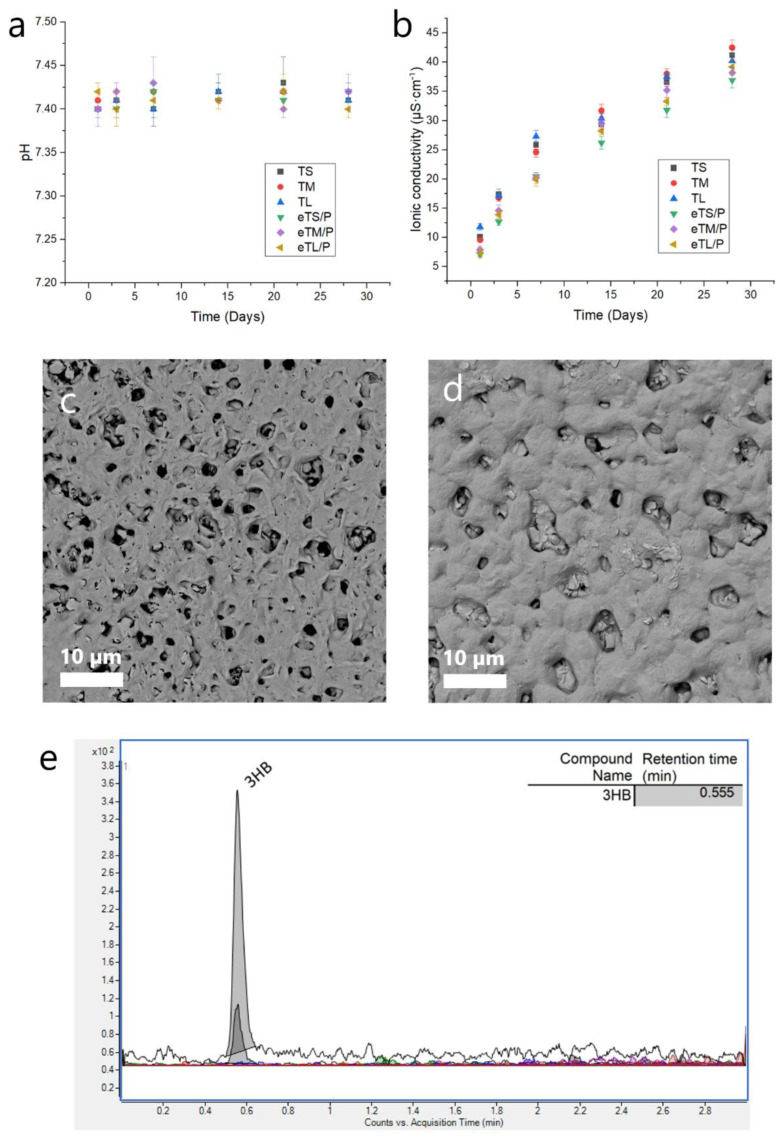
The changes in (**a**) pH of SBF during sample incubation and (**b**) ionic conductivity around samples incubated in distilled water. Morphology of the P(3HB) on the eTM/P scaffold (**c**) before and (**d**) after 60 days of incubation in distilled water. The results of the UHPLC-MS analysis of the sample after 60-day incubation concerning the presence of hydroxy acids (**e**).

The potential clinical implications of the implanted materials can be predicted by performing chemical stability and degradation studies in vitro. Therefore, a 28-day incubation was conducted for T and eT/P materials series. The studies revealed that scaffolds did not influence the pH of SBF over this time (Figure 10a). Similarly, the ionic conductivity of water in which the materials were incubated for 28 days increased marginally (Δ ≈ 40 µS∙cm^−^^1^ in the case of all specimens) (Figure 10b). After 60 days of incubation in water, the morphology of the surface of eTM/P was changed. Over this time, the surface of P(3HB) was smoothed and larger micropores appeared, suggesting surface erosion of the polymer coating (Figure 10c vs. Figure 10d). This was confirmed by UHPLC-MS analysis, which enabled to detect low quantities of (*R*)-3-hydroxybutyric acid—a monomer originating from the degradation of P(3HB) (Figure 10e). It is well known that 3-hydroxybutyric acid (3HB) is an organic acid naturally occurring within the bloodstream as one of the β-oxidation pathway metabolites. The slow release of 3HB over time can potentially nourish the regenerating tissue at the site of the implantation of a polymer-ceramic scaffold [45]. Additionally, it was shown that 3-hydroxybutyric acid and its derivative 3-hydroxybutyric methyl ester effectively reduce osteoporosis under both in vitro and in vivo studies [46], which suggests another prospective way of obtaining composites’ applications.

### 2.7. In Vitro Biological Studies

**Figure 11:**
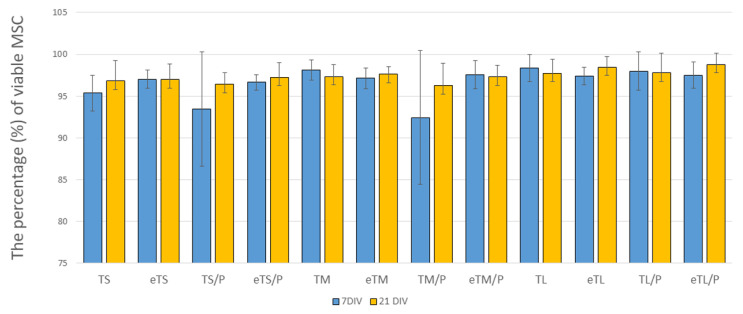
The mean percentage of viable MSC at 7 and 21 DIV. Test: One-way ANOVA with post hoc Bonferroni’s multiple comparisons test, alpha 0.05 (95% confidence interval).

The mean percentages of viable MSCs are presented in Figure 11. The data show that the mean cell viability was above 90% for all tested materials at 7 and 21 DIV. A slight decrease in MSCs viability has been observed in the case of cells growing on untreated T/P scaffolds at 7DIV (especially in the case of TS/P and TM/P); a similar effect has not been observed in the case of MSCs grown on eT/P scaffolds. Furthermore, on eT and eT/P scaffolds cell viability was over 95%, these values are comparable to the viability of MSCs on tissue culture polystyrene (TCPS), which indicates the lack of cytotoxicity of the studied materials. These results are compared to those obtained by other authors, indicating that both P(3HB) [15,45] and TCP [4] are not cytotoxic. Cell viability was high, irrespective of scaffolds’ pore size.

**Figure 12:**
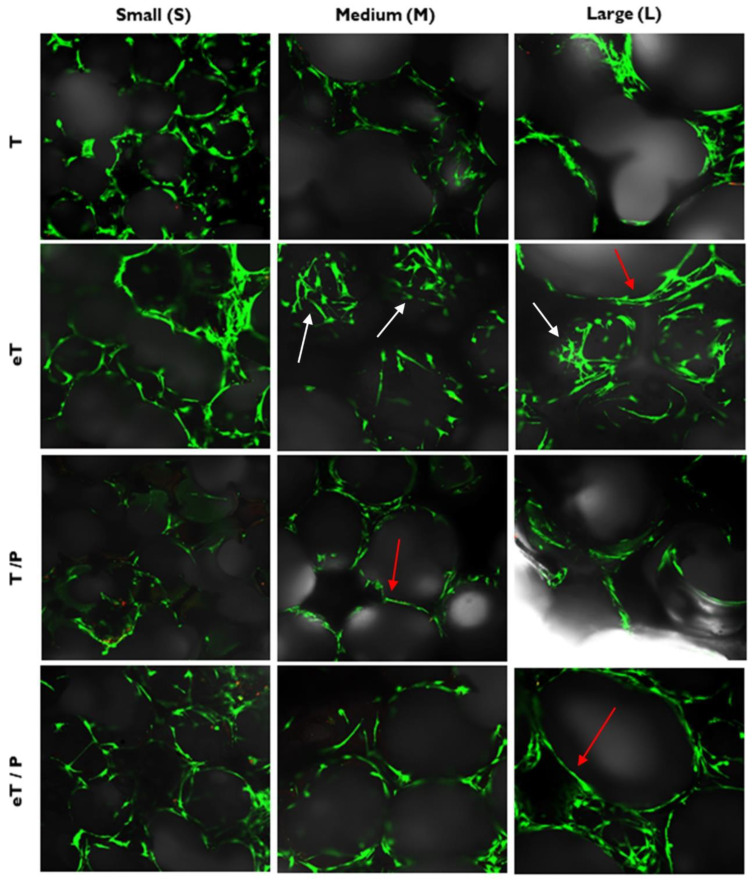
Growth of MSC cells on scaffolds. Live cells are green and dead are marked with red dots. Red arrows show cells growing on the edges of the pores and the white arrows show cells growing on the bottom and sides of the pores. Image captured under 10× objective.

**Figure 13:**
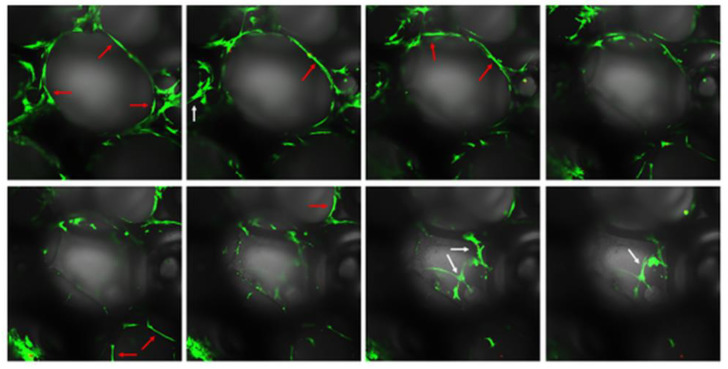
Z-stack of MSC cells growing on eT/M scaffold. Photographs were taken at different focal planes along the vertical z-axis (interval 40 µm), showing the location of the cells in the scaffold pore. Red arrows show cells growing on the edges of the pores and white arrows show cells growing on the bottom and walls of the pores.

No differences in cells’ growth behaviour for tested materials (i.e., T, eT, T/P, and eT/P scaffolds) were observed. In each case, cells grew on struts (pore edges, rims) and, penetrating the materials, overgrew the bottom and walls of the pores. The cells exhibited a fibroblast-like morphology, indicating good attachment to the scaffolds’ surfaces. According to the literature, porosity and pore size are critical values for MSCs growth and proliferation [47,48]. As mentioned above, the average pore size was 272 ± 79 µm, 503 ± 147 µm and 727 ± 229 µm for TS, TM and TL, respectively, and the scaffold coverage with P(3HB) did not significantly change these values. Further, pores above 300 µm in diameter promote bone regeneration and allow vascularization [49,50], so the developed scaffolds meet this criterion. Figure 12 and Figure 13 show that cells grew over the entire surface of the materials between the pores, with a tendency to occupy the edges of the pores-visible protrusions running along the edge line (red arrows), and also to cover the bottom and sides of the pores (white arrows). Nevertheless, it can be noticed in Figure 12 that there are more cells on the eT and eT/P materials compared to the untreated scaffolds. This may be due to the greater variety of pores and increased surface roughness in the materials after etching. Knychala et al. [51] claimed that tissue formation favours concave surfaces if compared with flat and convex regions. Further, researchers proved that faster cell invasion occurred in narrower pores.

**Figure 14:**
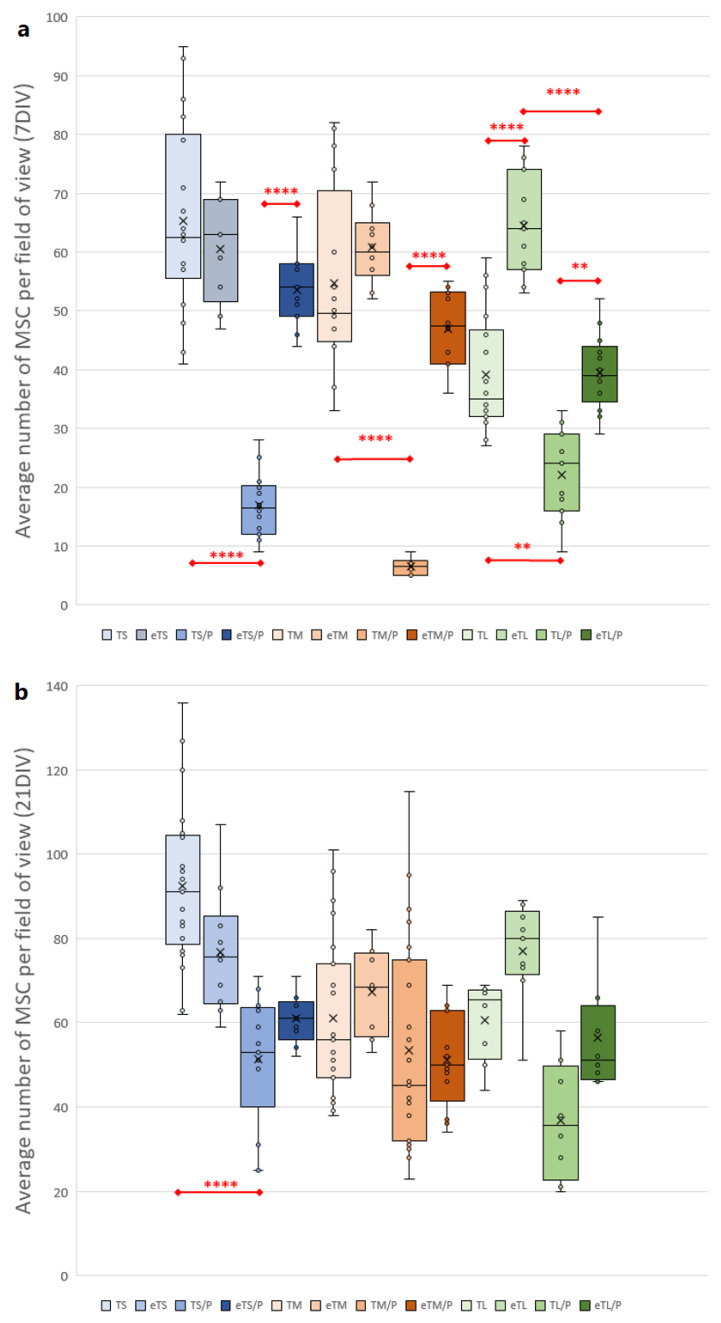
The average number of MSC cells growing on scaffolds per field of view at (**a**) 7 and (**b**) 21 DIV. Test: One-way ANOVA with post hoc Bonferroni’s multiple comparisons test, alpha 0.05 (95% confidence interval), Significant digits (*) for *p*-value * 0.0332, ** 0.0021, *** 0.0002, **** 0.0001. Box plot showing the median (horizontal line), X—mean value, the box covers values from the first to the third quartile and whiskers—total range.

A noticeable difference between the number of cells growing on the ceramic (T) and composite (T/P) scaffolds (Figure 12 and Figure 14) was observed. Infiltrating scaffolds with P(3HB) lead to the reduction of the number of growing cells, especially at the beginning of culture (7DIV). On TCP scaffolds with small pores (TS), an average number of cells was 65; on a corresponding scaffold covered with P(3HB), the number of cells was reduced to 17; on a scaffold with medium pores from 55 (TM) to 7 (TM/P), on a scaffold with large pores from 39 (TL) to 22 (TL/P) (all statistically significant). This reduction may be related to the fact that P(3HB) is a hydrophobic material [52]. Hydrophobicity may reduce initial cell access to the material surface, for example by preventing the flow of the medium with suspended cells through the pores into the scaffold. However, it does not cause worse growth or proliferation of MSCs between the 7th and 21st day of culture as the average number of cells increases, both in the case of T as well as T/P scaffolds (Figure 14). Moreover, cell proliferation was more noticeable on scaffolds covered with P(3HB). The differences in the average number of cells between ceramic and ceramic–polymer scaffolds diminished and ceased to be statistically significant, but were still visible. On the 21st day of the culture, these values were as follows: an average number of cells growing on scaffolds with small pores—92 (TS) and 51 (TS/P) (statistically significant), with medium pores—61 (TM) and 53 (TM/P), and large pores—61 (TL) and 37 (TL/P). Similarly, Shumilova et al. observed an increase in cell number during the culture on P(3HB) scaffolds and no cytotoxic effect of polymer on MSCs [53].

A higher average number of cells on the etched bioceramic scaffolds (eT) during the time of culture was observed. This was possibly due to an increase in the scaffold’s surface roughness after etching. Wang et al. [54], who applied double etching on titanium alloy, found that the surface roughness had a significant positive effect on cell proliferation. This was also supported by Giner et al. [55], who use double acid etching treatment in order to enhance the biological properties of dental implants. Furthermore, CA treatment led to the creation of micropores—called windows—present at the scaffold’s pore walls, which not only facilitate cell adhesion but also promote contact between MSCs growing in adjacent pores (both direct and *via* signal molecules). It should be highlighted that such micropores facilitate the paracrine and immunomodulatory action of MSCs, which is an important part of the regeneration process [56]. Moreover, initial issues with cell access to the hydrophobic P(3HB) surface in T/P composites diminished after the etching of the βTCP scaffolds. For all pore sizes, cells grew significantly better at 7DIV on etched scaffolds covered with P(3HB) (eT/P) than on the untreated ones (T/P). The average number of cells growing on eTS was 61, while 54 on the analogous P(3HB)-coated scaffolds. For medium and large pores, these values were respectively: 61 (eTM), 47 (eTM/P), 64 (eTL), 39 (eTL/P). At 21 DIV, these values were as follows: 77 (eTS), 61 (eTS/P), 67 (eTM), 51 (eTM/P), 77 (eTL), 56 (eTL/P). Our results demonstrate that the pre-treatment by citric acid etching of bioceramic scaffolds increased initial composites’ (eT/P) availability to cells, facilitating their adhesion. This is probably related to the microstructure of the eT/P composite after etching. SEM images showed βTCP grains protruding from the P(3HB) layer (Figure 3 eT/P and Figure 10c,d). Hydrophilic βTCP protrusions [5] may reduce general hydrophobicity and facilitate cell adhesion.

**Figure 15:**
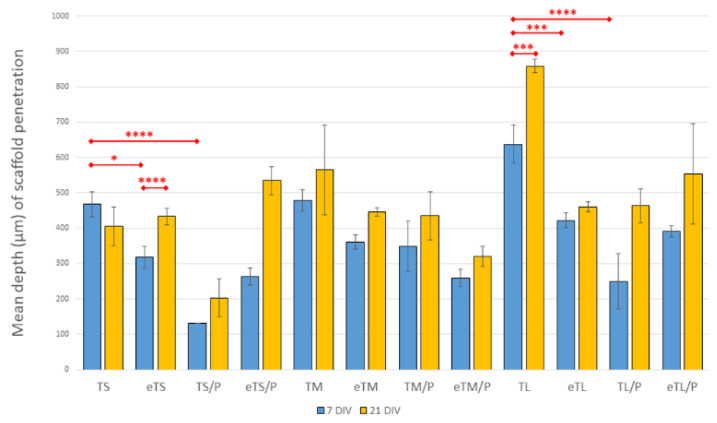
The mean depth (µm) of scaffold penetration (MSC overgrowth) at 7 and 21 DIV. Test: One-way ANOVA with post hoc Bonferroni’s multiple comparisons test, alpha 0.05 (95% confidence interval), Significant digits (*) for *p*-value * 0.0332, ** 0.0021, *** 0.0002, **** 0.0001, whiskers—standard deviation.

The average depth to which the cells penetrated scaffolds is presented in Figure 15. On the 7th day of culture in the case of T/P scaffolds, penetration was lower than on T scaffolds. However, in the case of eT and eT/P materials, these differences become less relevant. On the 21st day of culture, the penetration depth increased, which indicates that cells migrate into the scaffolds. In general, previous studies conducted within atmospheric oxygen levels (21%) showed that BM MSCs adhere, overgrow, and divide on PHA-based materials (including P(3HB)) [52,53,54] as well as on βTCP [57]. Our research shows that the combination of these two components also provides a good environment for adhesion, growth, and proliferation of MSCs in lowered physiological oxygen levels (5%). This demonstrates that the MSCs could attach, spread, and proliferate on the materials in all types of scaffolds, suggesting a positive cellular behaviour.

## 3. Materials and Methods

### 3.1. βTCP Synthesis and Powder Preparation

βTCP powder was synthesized by the wet precipitation method according to PL 190486 patent [58]. Briefly, calcium oxide (POCH, Gliwice, Poland) and 85% solution of phosphoric acid (POCH, Gliwice, Poland) were used as reagents (Ca/P = 1.5). After 48 h, the precipitate was centrifuged, dried, ground in a mortar, next in a ball mill (Retsch PM100), and sieved (<63 μm). The obtained powder was calcined at 900 °C, ground in an attritor mill for 4 h, and sieved to a grain size below 63 μm.

### 3.2. P(3HB) Synthesis

Poly(3-hydroxybutyrate) (P(3HB)) was produced by bacterial fermentation from glycerol (Orlen Południe S.A., Trzebinia, Poland) in the presence of NaCl (Chempur, Piekary Śląskie, Poland) at 45 °C using bacterial strain Zobellella denitrificans (Wilhelms-Universität Münster, Münster, Germany), as described previously [59]. The biomass after fermentation was lyophilized and then extracted with chloroform (Chempur, Piekary Śląskie, Poland). The resulting solution was filtered through activated charcoal (Merck, Warsaw, Poland) and 0.2 µm polytetrafluoroethylene (PTFE) filter (Avantor, Gdańsk, Poland). Next, the P(3HB) solution was concentrated on a rotatory evaporator (Heidolph Hei-VAP Industrial B, Heidolph Instruments GmbH & Co. KG, Schwabach, Germany), precipitated in an ice-cold methanol solution (Chempur, Piekary Śląskie, Poland), and dried in an oven (Binder FED400, Binder GmbH, Tuttlingen, Germany). The purified polymer was reconstituted in chloroform (5% *w*/*v*) for further experiments.

### 3.3. βTCP and Composites βTCP/P(3HB) Preparation

βTCP scaffolds were obtained by a foam replication method using three types of polyurethane matrices with different pore sizes (Bulpren S type 31048, 28089 and 28133). Cubic foams with an edge length of ~10mm were impregnated in the slurry composed of βTCP powder, distilled water, Dispex^®^ AA4040 (BASF, Arnhem, The Netherlands) and methylcellulose (Fluka, Buchs, Switzerland). Next, the specimens were dried and sintered at 1150 °C. Ceramic–polymer composites were prepared in two different routes. In the first method, the obtained ceramic specimens were infiltrated with 5% (w/v) P(3HB) chloroform solution and dried at room temperature for 7 days. In the second one, βTCP scaffolds were firstly treated with 5% (*w*/*v*) aq. Solution of citric acid (POCH, Gliwice, Poland) for 3 min, thoroughly washed with distilled water, dried at 80 °C for 24 h, and then the pre-treated ceramic sinters were infiltrated with 5% (*w*/*v*) P(3HB) chloroform solution and dried at room temperature for 7 days. The description of the obtained materials is presented in Table 1. To study the phase composition of the composite and the thickness of the P(3HB) layer, the βTCP discs were prepared using a hydraulic press (Graseby Specac, Orpington, UK). βTCP powder was pressed under the pressure of 100 MP, sintered at 1150 °C, treated in the same manner with citric acid, and covered with P(3HB).

### 3.4. Phase Composition

The XRD patterns were collected using a Bruker D2 Phaser diffractometer (Bruker, Billerica, MA, USA) with CuKα radiation (1.54 Å), an electron beam energy of 30 kV, and 10 mA intensity. The scans were recorded over the 2θ range from 10° to 40° at 0.04° intervals with a scanning speed of 2.5°∙min^−1^. Crystallographic identification was performed by comparing the experimental patterns to the Joint Committee on Powder Diffraction Standards (JCPDS): βTCP (00-055-0898) and CDHA (01-074-9779).

### 3.5. Thermal Analysis

The thermogravimetry (TG) and differential scanning calorimetry (DSC) measurements were carried out in flowing synthetic air (50 mL∙min^−1^) using a Simultaneous Thermo Analyzer (STA) TG-DSC NETZSCH STA 449 F5 Jupiter (Netzsch, Selb, Germany). As a reference substance, an empty α-Al_2_O_3_ crucible was used. Samples were heated from 40 °C to 500 °C with a heating rate of 10 °C⋅min^−1^ in the Al_2_O_3_ crucible.

### 3.6. Microstructure

The microstructure observations of the obtained materials were performed using a Phenom Pure (Gen. 5, Thermo Fisher Scientific, Waltham, MA, USA) scanning electron microscope. Images were captured at an accelerating voltage of 10 kV with a backscattered electron detector. Prior to microscopic observations, samples were sprayed with a thin layer of gold. The average pore size of the materials was calculated by measuring 100 pores and the results were presented as the average value ± standard deviation.

### 3.7. Porosity

The porosity of the scaffolds was determined by Archimedes’ technique with the use of analytical balance Radwag WPA 60/C along with the set (table and container) for hydrostatic weighing. Measurements were made in sextuple and the results were presented as the average value ± standard deviation.

### 3.8. Wettability and Surface Free Energy Measurements

The wettability of the flat surfaces (discs prepared in the same manner as in XRD measurements) was determined by measuring the water contact angle using a DSA 25 goniometer (Kruss, Hamburg, Germany). The test was performed at room temperature (~23 °C) by placing a drop of deionized water on the materials. The surface free energy was determined using an Owens-Wendt method. Measurements were made in sextuple and the results were presented as the average value ± standard deviation.

### 3.9. Compressive Strength

The compressive strength of the scaffolds was measured using a universal testing machine Instron 3345 (Instron, Norwood, MA, USA) under a head speed of 1 mm⋅min^−1^. The mechanical properties of ≥10 cubic samples with an edge length of about 8 mm were evaluated and the results were presented as the average value ± standard deviation.

### 3.10. Chemical Stability and Degradation Studies In Vitro

To determine the chemical stability in vitro of the materials, bioceramic scaffolds (TS, TM and TL) and composites (eTS/P, eTM/P, eTL/P) were incubated in distilled water or simulated body fluid (SBF) prepared according to the procedure described by Kokubo [60]. Specimens were placed in sterile polypropylene containers, filled with water or SBF in a ratio of 100 mL per 1 g of the sample and kept in an incubator at 37 ± 0.5 °C. The pH of SBF and the ionic conductivity of the water were measured after 1, 3, 7, 14, 21, and 28 days of incubation using a pH-meter/conductometer Mettler Toledo Seven Compact Duo (Mettler Toledo, Columbus, OH, USA). The measurements were made in triplicate. Results were presented as the average value ± standard deviation. Furthermore, to asses degradation products originated from the polymer, composite samples (eTM/P) were incubated in distilled water at 37 °C in polypropylene (PP) containers for 60 days. Supernatants (1 mL) were filtered and analysed on Agilent 1290 Infinity System with an automatic autosampler and MS Agilent 6460 Triple Quad Detector (Agilent, Santa Clara, CA, USA) equipped with Agilent Zorbax Eclipse Plus C18 column (2.1 × 50 mm, 1.8 µm) (Agilent, Santa Clara, CA, USA) similarly to previously reported method [35]. Briefly, samples were developed on the column at 30 °C at a flow rate of 0.5 mL∙min^−1^ and with gradient elution of solvent A (0.1% *v*/*v* formic acid in water) and solvent B (0.1% *v*/*v* formic acid in acetonitrile) as follows: 0.00 min (50% A/50% B) to 1.90 min (10% A/90% B) to 1.91 min (50% A/50% B) to 2.60 min (50% A/50% B). The injection interval was 2.6 min. MS Agilent 6460 Triple Quad tandem mass spectrometer with Agilent Jet Stream ESI interface (Agilent, Santa Clara, CA, USA) was used in negative ion mode. Nitrogen at a flow rate of 10 L∙min^−1^ was used as the drying gas and for collision-activated dissociation. Drying gas and sheath gas temperatures were set to 350 °C. The capillary voltage was set to 3500 V, whereas the nozzle voltage was set to 500 V. Elution profiles were monitored in a scan range of 50–1000 m/z first, to determine main peaks. Next, main degradation compounds were monitored in multiple reaction monitoring mode (MRM) with the transitions, polarity, fragmentor (F), and collision energies (CE). MassHunter software (Agilent, Santa Clara, CA, USA) was used for HPLC-MS system control, data acquisition, and data processing.

### 3.11. In Vitro Biological Studies

For biological studies, materials were sterilized with ethylene oxide. Biocompatibility evaluation of the scaffolds was performed using human bone marrow-derived mesenchymal stem cells (BM hMSCs). Before the experiment, the human Mesenchymal Stem Cells (Lonza, PT-2501, Walkersville, MD, USA) were grown in a culture flask (Nunc, 75T, Thermo Fisher Scientific, Waltham, MA, USA), in MSCGM medium (Lonza, PT-3001, Walkersville, MD, USA). Cells were incubated at 37 °C with 5% oxygen tension, 5% carbon dioxide, and 95% humidity. The culture medium was changed three times a week. Before seeding the cells, scaffolds were rinsed and soaked in PBS (12 h), culture medium (1 h) and then placed in 5 mL Eppendorf tubes. Cells were trypsynised using Trypsin/EDTA mixture (Lonza, CC-3232, Walkersville, MD, USA), centrifuged and resuspended in MSCGM medium. Then, the scaffolds were placed in Eppendorf tubes, covered with cell suspension, and incubated for 24 h. Further, the scaffolds with the medium were transferred to standard 6-well culture plates (Nunc, Thermo Fisher Scientific, Waltham, MA, USA) and cultured for 3 weeks. The MSC viability and growth in the 7th and 21st day in vitro (DIV) culture were assessed. Cell viability was assessed using LIVE/DEAD™ Viability/Cytotoxicity Kit, for mammalian cells (ThermoFisher, Waltham, MA, USA), which contains two reagents green-fluorescent calcein-AM (staining of live cells) and red-fluorescent ethidium homodimer-1 (dead cells). Cells were placed in calcein-AM/ethidium homodimer-1/DPBS solution according to the producer’s instructions 20 min before the microscopic observations and then visualised using LSM 710 NLO confocal microscope (Zeiss, Jena, Germany). Live (green- calcein-AM) and dead (red-ethidium homodimer-1) cells were counted, and then the number of live cells was divided by the number of all cells and multiplied by 100.

### 3.12. Statistics

Statistical analysis was performed using one-way analysis of variance (ANOVA) and post hoc Tukey HSD multiple comparisons. Statistically significant differences were indicated by * (*p* ≤ 0.01). For cell studies (cell average number, viability and penetration) one-way analysis of variance (ANOVA) and post hoc Bonferroni multiple comparisons test were used (alpha 0.05). Statistically significant differences were indicated by digits *. For *p*-value *p* < 0.0332 (*), *p* < 0.0021 (**), *p* < 0.0002 (***), *p* < 0.0001 (****).

## 4. Conclusions

In this study, macroporous bioceramic βTCP and composite βTCP/P(3HB) scaffolds with different pore sizes were successfully obtained by the polyurethane sponge replica method followed by polymer infiltration. To enhance the attachment of the biopolymer to the βTCP surface, pre-treatment of the ceramic sinters with citric acid was performed. The etching affected the microstructure of the ceramic scaffolds creating micropores and rougher surfaces, resulting in improved adhesion between the composite components at the ceramic/polymer interface. The thermogravimetry measurements revealed that the polymer content in the composites was ~3.0 wt%. The endothermic melting and decomposition reactions of P(3HB) were observed. The P(3HB) coating did not significantly influence the average pore size (from 312 ± 97 µm to 670 ± 352 µm) and total porosity (from 67.0 ± 2.0 to 70.9 ± 1.7 vol%) of the obtained materials. Improved attachment of the polymer to the etched βTCP scaffolds had a positive impact on their compressive strength (up to 4.5 ± 0.5 MPa) and durability due to the crack bridging by P(3HB). Obtained materials were chemically stable in vitro. UHPLC-MS analyses confirmed the degradation of P(3HB) to 3-hydroxybutyric acid after 60 days of composite incubation in water. According to the literature, this compound can potentially nourish surrounding tissues and reduce osteoporosis, which broadens the applicability of developed materials in bone tissue engineering.

Furthermore, it has been shown that the mesenchymal stem cells cultured on the obtained scaffolds in a low oxygen concentration (5%)—which mimic the conditions that would be found in damaged bone in a living organism during regeneration—adhere, spread, and proliferate on the materials’ surface, providing cell-friendly environment. Moreover, scaffolds with all pore sizes enabled cell migration and sufficient flow of the culture medium (supply of nutrients, oxygen, and removal of metabolic products) ensuring high cell viability. It has been demonstrated that βTCP pre-treatment led to the creation of micropores which promote contact between MSCs growing in adjacent pores. In addition, in composites with etched βTCP, hydrophilic ceramic protrusions reduced surface hydrophobicity (water contact angle was equal 99° ± 8° for βTCP/P(3HB) vs. 67° ± 5° for eβTCP/P(3HB)) and facilitated cell adhesion. Further assessment of the obtained composites will be focused on in vivo studies.

## Figures and Tables

**Table 1:** The description of the studied materials.

Material Symbol	Description
TS	βTCP scaffolds prepared using Bulpren S 31048 polyurethane sponges (small pores)
TM	βTCP scaffolds prepared using Bulpren S 28089 polyurethane sponges (medium pores)
TL	βTCP scaffolds prepared using Bulpren S 28133 polyurethane sponges (large pores)
TS/P	TS scaffolds covered with P(3HB)
TM/P	TM scaffolds covered with P(3HB)
TL/P	TL scaffolds covered with P(3HB)
eTS	TS scaffolds treated with a citric acid solution
eTM	TM scaffolds treated with a citric acid solution
eTL	TL scaffolds treated with a citric acid solution
eTS/P	eTS scaffolds covered with P(3HB)
eTM/P	eTM scaffolds covered with P(3HB)
eTL/P	eTL scaffolds covered with P(3HB)

## Data Availability

The raw/processed data required to reproduce these findings can be obtained from the corresponding author upon reasonable request.
